# Converging evidence for enduring perceptions of low social status in individuals in remission from depression

**DOI:** 10.1016/j.jad.2021.07.083

**Published:** 2021-11-01

**Authors:** Julia A. Gillard, Siobhan Gormley, Kirsty Griffiths, Caitlin Hitchcock, Tim Dalgleish, Jason Stretton

**Affiliations:** a Medical Research Council Cognition and Brain Sciences Unit, University of Cambridge, 15 Chaucer Road, Cambridge CB2 7EF, United Kingdom; b Cambridgeshire and Peterborough NHS Foundation Trust, United Kingdom

**Keywords:** Social status, Remitted depression, Major depressive disorder, Community sample, Case-Control

## Abstract

**Background:**

: The risk of depressive relapse and recurrence is associated with social risk factors that may be amplified by a submissive socio-cognitive profile.

**Methods:**

: In Study 1 we aimed to identify perceptions of low social status in a community sample (*N* = 613) with a self-reported history of mental health difficulties (*n* = 232) and, more specifically in Study 2 (*N* = 122), in individuals in clinical remission from depression (*n* = 18), relative to a never-depressed control group (*n* = 64), and relative to a group experiencing a current depressive episode (*n* = 40).

**Results:**

: In Study 1, a total of 225 of the 232 participants in the self-reported mental health difficulties group opted to provide further information regarding their mental health history, of whom 153 (68%) reported a history of anxiety, 168 (74.7%) reported a history of depression, and 13 (5.8%) reported an unspecified mental health history. Elevated depressive symptoms were associated with perceptions of low social status which significantly differed between individuals with and without a self-reported history of mental health difficulties. In Study 2 we found enduring perceptions of low social status in remitted depressed individuals.

**Limitations:**

: We were unable to discern between historical or current clinical diagnosis in the community sample of Study 1, as we were reliant on self-report. We were unable to explore the effects of medication or causal relationships between depressive symptoms and social status as the studies were cross-sectional in nature.

**Conclusions:**

: These findings suggest that evolutionarily rooted socio-cognitive profiles could impact affiliative processes and may confer increased vulnerability to future depressive episodes.

## Highlights

• Perception of low social status is a risk factor for depression.
• Investigated social status in a community sample and a case control design.
• BDI score associated with perceptions of low social status in a community sample.
• Perceptions of low social status endured in clinical remission from depression.

## 1 General introduction

Depression is a common psychiatric condition marked by a heightened risk of relapse and recurrence, amplified in the presence of social risk factors. Indeed, aside from persistent low mood and anhedonia, depression has long been characterised by maladaptive social functioning attributed to underlying socio-affective and cognitive difficulties (Hirschfeld et al., 2000; Kennedy et al., 2007; Ladegaard et al., 2014). Perceived or actual social rejection is posited as one of the stronger risk factors for developing depression, with early interpersonal difficulties viewed as potential psychosocial antecedents of later depression (Heim and Binder, 2012; Luterek et al., 2004; Slavich and Irwin, 2014; Slavich et al., 2010; van Harmelen et al., 2014, 2010).

The Beck Depression Inventory (BDI) is commonly drawn on for the identification of current or residual symptoms of depression and is well-suited for assessing the broad symptom severity of depression (Beck et al., 1997; Lasa et al., 2000; Osman et al., 2002; Storch et al., 2004). However, measures of social functioning are seldom included amongst lists of such core symptoms, such as persistent low mood and loss of interest or pleasure in activities, and are rarely employed as primary functional treatment outcomes, or examined throughout the course of treatment (Hirschfeld et al., 2000; Kennedy et al., 2007; Park et al., 2014; Renner et al., 2014). This is despite evidence for long-term difficulties in social and physical functioning across various social roles and domains even after remission or recovery from depressive disorders (Rhebergen et al., 2010). Thus, rate of relapse and recurrence may improve if we can gain a better understanding of the social and cognitive mechanisms involved in depression.

Specific social and cognitive mechanisms pertaining to social functioning can also be conceptualised within evolutionarily derived theoretical models focusing on the role of social status; for example, Social Rank Theory (Allan and Gilbert, 1997; Gilbert et al., 2007), and the Social Risk Hypothesis of depressed mood (Allen and Badcock, 2003; Badcock et al., 2017). Indeed, one of the core social challenges is how individuals compete for resources and status that regulate resource acquisition. The outcome of those competitions have been shown to have an impact on physiological systems and subsequent behaviour (Weirs et al., 2016). According to these theories, depressed mood developed as an evolved adaptive coping mechanism to reduce agonistic social interactions when status is critically low, with corresponding elevated risk of rejection or ejection from the social group. It is important for individuals who are in subordinate status to be wary of those who are above them and regulate their explorative and resource seeking behaviours. Individuals who are of lower status have to be cautious of those with higher status at the same time as gauging opportunities to successfully improve their own rank. In such circumstances, accepting social defeat (i.e. loss or rejection) and maintaining a low social rank is arguably evolutionarily advantageous, relative to challenging the status quo and risking a further, and potentially critical, loss of status. Such acceptance involves appeasing aggression or threats by a more dominant others and inhibiting socially risky behaviour, in the context of information signaling defeat, inferiority or low social rank (Allen and Badcock, 2006), in order to maintain inclusiveness in the group.

Social Rank Theory proposes some form of internal gauge as the cognitive device which evaluates current trajectories of social defeat or success based on incoming social cues. Similarly, the Social Risk Hypothesis argues that depressed mood is the result of a critically low Social Investment Potential (SIP) (Allen and Badcock, 2003). SIP represents the balance of an individual's social value or benefit to others, relative to their social burden to others (e.g. their need for a share of valuable resources). Clinical depressed mood arises when low SIP becomes entrenched, with no obvious or available behavioural strategies to repair status while minimising social risk. Behaviourally, this profile is akin to learned helplessness (Price et al., 1994) and is one path of many to clinically depressed mood (see Akiskal and McKinney, 1973 for review). In the context of social status, this pathway is initiated by repeated social defeat and feelings of entrapment (Taylor et al., 2011; Wetherall et al., 2019). Thus, in those with a history of prior depression that is now in remission, enduring perceptions of low social status may point towards a learned and consolidated ‘lower rank mindset’. This mind-set, once initiated, may then switch the internal gauge of social rank or critically low SIP to a maladaptive state of inferiority, even in the absence of current depressed mood.

The process of social comparison encapsulates this universal tendency for monitoring our social position within a hierarchy (Allan and Gilbert, 1995; Giacolini et al., 2013; Gibbons and Buunk, 1999; Tajfel, 1978; Terol et al., 2015). It is argued that such comparison provides the mechanism by which the internal gauge or SIP motivates behaviour, including greater submissiveness and involuntary subordination, i.e. feeling unable to escape from a defeating situation or unable to accept the defeat (Sturman, 2011). These behavioural strategies are employed to maintain a stable (low) social position within the hierarchy rather than risk expulsion (Allan and Gilbert, 1997; Baumeister and Leary, 1995; Gilbert et al., 2007; Leary, 2004).

Previous work aimed at identifying systemic biases in social processing have led to the development of an array of self-report measures targeting known areas of social difficulty in depression. These measures include the Social Comparison Scale (SCS), assessing for feelings of low rank (Allan and Gilbert, 1995), the Submissive behaviour Scale (SBS) (Allan and Gilbert, 1997), as well as the (insecure and secure) Striving to Avoid Inferiority Scale (SAIS) (Gilbert et al., 2007), and Involuntary Subordination Questionnaire (ISQ) (Sturman, 2011). These measures are central to understanding social functioning, with self-perceptions of low social rank and submissive behaviour found to correlate with a range of clinical disorders, including depression and anxiety problems (Wood and Irons, 2015), as well as clinical manifestations, including paranoid thoughts (Freeman et al., 2005), low self-esteem and perceived social stigma (Paterson et al., 2012).

Insecure striving to avoid inferiority is motivated by fear and avoidance of rejection or criticism for falling behind within the social dynamic, in contrast to secure non-striving (e.g. feeling socially acceptable and valued regardless of interpersonal success) (Gilbert et al., 2007). Insecure striving is associated with submissiveness and depressive symptoms in healthy and depressed adult populations (Gilbert et al., 2007; Gilbert et al., 2009). In young adults, insecure striving is correlated with self-harm, perceptions of low rank and depressive symptoms (Ferreira et al., 2013; Williams et al., 2009), while non-striving and acceptance are negatively associated with anxiety, fear of rejection and depressive symptoms (Bellew et al., 2006).

The measure of involuntary subordination – the ISQ – was developed to encompass and supersede previous measures investigating negative social comparison, feelings of inferiority, and submissiveness (Sturman, 2011). Involuntary subordination may be adaptive in species, including humans, which compete for resources, acting as a mechanism to switch off conflict behaviours when loss is imminent (thus saving an organism from harm). However, in humans, major depression is theorised to occur when involuntary subordination becomes prolonged (Gilbert, 2000; Sturman et al., 2015). The ISQ predicts short-term changes in social anxiety symptoms, and involuntary subordination has been shown to mediate the relationship between defeat and depressive symptoms in healthy undergraduates, as well as being highly correlated with self-criticism, neuroticism, and low self-esteem (Sturman, 2011; Sturman et al., 2015). However, it has not been explicitly validated in currently depressed or remitted depressed samples, despite defeat and entrapment being associated with symptoms of depression, anxiety, suicidality, suicidal ideation and post-traumatic stress disorder (PTSD) (Li et al., 2016; Siddaway, 2013).

The high rate of relapse and chronicity in individuals with a history of depression represents a significant social and clinical problem, with recurring depressive episodes further contributing to the economic and mental health burden (Greenberg et al., 2015; Paykel, 2008). However, to date, few studies have examined social processing strategies using measures such as those described above in currently depressed samples, let alone in remitted depressed participants, despite the significant overlap across psychopathologies (Kennedy and Adolphs, 2012) and consistent evidence that social processing significantly predicts future symptoms. Implementing measures of social processing outlined above in a large community sample, together with measures of depressive symptomatology allows us to directly test the hypotheses of Social Rank Theory (Allan and Gilbert, 1997; Gilbert et al., 2007) concerning perceived subjective low social status and low mood. Furthermore, using the same measures in samples of currently depressed and remitted depressed individuals allows us to test the predictions of the Social Risk hypothesis (Allen and Badcock, 2003). Specifically, we tested how perceived social status and subsequent defensive behaviours may continue to negatively impact the ability to form or maintain positive social relationships within social hierarchies, even in the absence of marked current depressive symptoms.

Identifying enduring maladaptive social processing strategies would therefore point towards potential risk factors of relapse from within the social domain specifically. This presents an important gap that needs addressing. Thus, in two studies presented here, we aimed to characterize the profile of perceptions of social status in individuals with low mood and depression. The first study investigated the relationship between low mood and perceptions of subjective social status in a community sample, while the second study more specifically examined socio-affective behaviours in individuals either with a current diagnosis of clinical depression or in remission from depression, relative to never-depressed individuals, using a case-control design.

## 2 Study 1: exploring the relationship between symptoms of depression and processing of social status in a community sample

### 2.1 Aims

Study 1 explored the relationship between self-reported acute depressive symptomatology and perceptions of subjective social status in an adult community sample. We hypothesized that higher levels of symptomatology would be positively correlated with perceptions of low social status and submissive behaviour in line with the predictions of Social Rank theory (Gilbert and Allan, 1998) and the Social Risk Hypothesis of Depression (Allen and Badcock, 2006). We also further predicted that those individuals with a self-described history of psychological problems would perceive themselves as being of lower social status and report greater submissive behaviour relative to those participants with no self-reported history of mental health difficulties.

### 2.2 Methods and materials

#### 2.2.1 Participants

Six hundred and thirteen participants (age range 18–65 years) were recruited using the online platform Prolific (www.prolific.co), an established portal for online subject recruitment, which explicitly caters to researchers. It combines good recruitment standards with reasonable cost, explicitly informs participants that they are recruited for participation in research (Palan and Schitter, 2018) and has been validated for use in research of clinical populations (Shapiro et al., 2013). All participants were from English-speaking countries (UK, Ireland, USA, Canada, & Australia) and were asked to fill out a series of questionnaires and provide demographic details. A multivariate outlier analysis to remove any participants who were not paying attention, identified via a series of periodically placed catch questions i.e. “Are you paying attention? (Please click I'm not paying attention)” or did not answer questions with suitable responses, left 600 participants for analysis.

All participants were asked to self-identify whether they had ever experienced psychological problems in the past. Based on this information, the sample was split into a healthy control group (those who reported no prior mental health difficulties) of 368 individuals (233 females, 42.26 ± 12.79; 133 males, 41.87 ± 13.74; 1 other, 31). Our self-identified mental health difficulties group comprised 232 participants (164 females, 40.7 ± 12.87; 65 males, 38.1 ± 14.82; 3 others, 35.67 ± 24.54). A total of 225 of the 232 participants in the self-reported mental health difficulties group opted to provide further information regarding their mental health history, of whom 153 (68%) reported a history of anxiety, 168 (74.7%) reported a history of depression, and 13 (5.8%) reported an unspecified mental health history. Comorbidity was reported in 118 participants (52.4%) with a median of two comorbid disorders (range = 3). Supplementary Table 1 (see Supplementary Materials) shows the demographic information for the whole sample, and for the self-reported no-mental health difficulties group and mental health difficulties group. Chi-squared analysis showed no significant associations between group and nationality, ethnicity, employment status or education. There was a small significant association between group and gender (χ^2^ (2) = 6.24, *p* = 0.04) and a borderline significant difference in age between groups (F [1, 593] = 3.74, *p* = 0.05, η _p_^2^ = 0.006).

#### 2.2.2 Social and affective measures

##### 2.2.2.1 Beck Depression Inventory(BDI-II:Beck et al., 1997)

The BDI-II is a 21-item self-report measure assessing depressive symptomatology including low mood over the previous two weeks. It is one of the most widely used instruments for measuring the severity of depression symptomatology with good internal consistency, test-retest reliability and convergent validity with standardised clinician assessments (Beck et al., 1997), enabling comparison across studies. As data collection was online for Study 1, we removed Question 9 (“I don't have any thoughts of killing myself”) as part of our safeguarding procedures, creating a 20-item measure.

##### 2.2.2.2 Interpersonal Sensitivity Measure (IPSM) (Boyce and Parker, 1989)

The IPSM is a 36-item measure assessing sensitivity to the interpersonal behaviour of others, to social feedback, and to (perceived or actual) negative evaluation by others (e.g. *“I feel insecure when I say goodbye to people”; “I worry about what others think of me”; “I am always aware of how other people feel”*). The 36 items are completed on a 4-point Likert-type scale (1= ‘very unlike me’, 2=’moderately unlike me’, 3=’moderately like me’, 4=’very like me’). The IPSM generates a total score as well as five sub-scale scores: ‘interpersonal awareness’; ‘need for approval’; ‘separation anxiety’; ‘timidity’; and ‘fragile inner-self’. Internal reliability in depressed clinical and non-clinical groups is good: Cronbach's alphas of 0.86 and 0.85, respectively (Boyce and Parker, 1989) and were replicated in a sample of patients with Major Depressive Disorder, with significant relationships between the subscale and total scores of the IPSM and greater rejection sensitivity (Luty, Joyce, Mulder, F. Sullivan, & McKenzie, 2002). Stability was indicated by a six-week retest reliability of 0.70 in an unselected student sample (Boyce and Parker, 1989). Moderate to large correlations with neuroticism (*r* = 0.66), self-esteem (*r* = 0.39), and a small correlation with emotional arousability (*r* = 0.11) in social anxiety disorder highlight the convergent and divergent validity of the IPSM (Harb et al., 2002).

##### 2.2.2.3 Involuntary Subordination uestionnaire (ISQ) (Sturman, 2011)

The ISQ is a 32-item questionnaire consisting of a series of statements that reflect how people may feel about themselves, and is specifically designed to interrogate involuntary subordination, indexing tendencies of feeling stuck (entrapment), defeated, inferior, and seeing the self as submissive (e.g. *“I let others criticise me or put me down without defending myself”, “I can see no way out of my current situation”*). Individuals are asked rate the degree to which they agree with each of the statements as they relate to themselves on a 5-point Likert-type scale (1 = ‘Strongly Disagree’ to 5 = ‘Strongly Agree’). In healthy undergraduates, scores on the involuntary subordination scale were found to predict short-term changes in social anxiety symptoms and mediate the relationship between defeat and depressive symptoms (Sturman et al., 2015). Previously, ISQ at baseline predicted depressive symptoms, social anxiety, and social phobia over time (*r* = 0.41, *p* < 0.001) and demonstrated high levels of internal reliability (alpha = 0.95) and test–retest reliability (*r* = 0.75) based on 83 participants who completed the ISQ a second time after 9 weeks (Sturman, 2011). The ISQ has also been used with PTSD populations (Siddaway, 2013), and amongst men who have sex with men (Li et al., 2016), but not hitherto with depressed samples.

##### 2.2.2.4 Striving to Avoid Inferiority Scale part I (SAIS-I) (Gilbert et al., 2007)

Part I of the SAIS is a 31-item scale to measure beliefs about striving to compete to avoid inferiority (e.g., *‘If I don't strive to achieve I'll be seen as inferior to other people’, ‘People who can't compete are seen as weak’*) and feelings of acceptance by others whether one succeeds or fails (e.g., *‘Others will accept me even if I fail’, ‘If I make mistakes, I know other people will still like me’*). These two sets of beliefs correspond to the ‘insecure striving’ and ‘secure non-striving’ subscales, respectively. Participants are asked to rate statements describing how they think and feel about the need to strive and compete in life responding on a five-point Likert scale of 0 = ‘never’ to 4 = ‘always’. The insecure striving and secure non-striving subscales are negatively correlated (*r* = −0.51) and demonstrate good test-retest reliability of 0.84 for ‘insecure striving’ and 0.69 for ‘secure non-striving’ and Cronbach's alphas of 0.92 for ‘insecure striving’ and 0.87 for ‘secure non-striving’ in a sample of 207 undergraduate students (Gilbert et al., 2007).

##### 2.2.2.5 Submissive Behaviour Scale (SBS) (Allan and Gilbert, 1997)

The Submissive Behaviour Scale consists of 16 examples of submissive behaviour, in which a series of statements describes how people act and feel about social situations (e.g. *“I agree that I am wrong even though I know I'm not”, “I let others criticise me or put me down without defending myself”*). Participants are asked to rate the degree to which each example applies to them on a five-point Likert Scale from 0 = Never to 4 = Always. The SBS has good internal reliability, with a Cronbach's alpha of 0.89, and a four-month test-retest reliability of *r* = 0.84 with an undergraduate student population (Gilbert et al., 1996). The SBS has been used in studies examining social comparison (social ranking) and evolutionary theory (Gilbert and Allan, 1998), studies of shame (Gilbert et al., 1994), and depression, with submissive behaviour strongly associated with depressive symptoms (*r* = 0.58), and significant group differences relative to a Never-Depressed group in a sample of 50 in-patient depressed individuals (O'Connor et al., 2002).

##### 2.2.2.6 Social Comparison Scale (SCS) (Allan and Gilbert, 1995)

The SCS is an 11-item scale designed to measure self-perceptions of social rank and relative social standing. This scale consists of 11 constructs and participants are required to make a global comparison of themselves in relation to other people and to rate themselves on a ten-point scale (e.g. ‘*In relationship to others I feel*: Incompetent 1 2 3 4 5 6 7 8 9 10 More competent.*’*). The 11-items cover judgements concerned with rank, attractiveness and how well the person thinks they ‘fit in’ with others in society. Low scores indicate feelings of inferiority and general low-rank self-perceptions. The SCS has been found to have good reliability, with scale reliabilities of alpha= 0.88 in out-patients with anxiety and depression, relative to 0.91 in Never-Depressed participants (Allan and Gilbert, 1995, 1997), and 0.89 in an eating disordered sample (Ferreira et al., 2013). Previous work using the SCS suggested that self-perceptions of lower rank resulting from social comparisons are associated with greater feelings of depression and low mood (O'Connor et al., 2002; Wood and Irons, 2015).

#### 2.2.3 Procedure

Participants were administered the battery of questionnaires on the Prolific Academic website in the same order. Participants were reimbursed for their time and the study was carried out in accordance with the Declaration of Helsinki and Good Clinical Practice and approved by the Cambridge Psychology Research Ethics Committee (PRE: 2017.083).

#### 2.2.4 Data analysis

Missing data within and across different measures constituted less than 5% of the overall data, thus considered to be missing at random and the univariate ANOVAs therefore implemented listwise deletion, followed by a missing value analysis using EM (expectation-maximisation) (Dong & Peng, 2013). This method assumes a distribution for the partially missing data and bases inferences on the likelihood under that distribution, considering the conditions under which missing data occurred.

In order to examine the relationship between mood and social status we applied Pearson correlations between BDI-II and all social measures across the entire sample. Significance was assessed using *p*-value <0.05 and gender was included as a potential confound. Secondly, in order to explore the impact of self-reported mental health difficulties, we carried out a series of univariate ANCOVAs for the battery of affective and social measures with group as the between-group factor and gender and age as co-variates across all analyses. Although we have several measures assessing a similar construct within the social domain (see Supplementary Table 3) we chose not to employ any data reduction techniques on the social measures for two specific reasons. Firstly, the ISQ was developed by using a factor analysis including the SBS and SCS, thus precluding its use in any further data reduction. Secondly, there is little validation data available on the independent social measures included in this study from large community samples.

## 3 Results

### 3.1 Whole sample correlations between BDI and each social measure individually

**Fig. 1:**
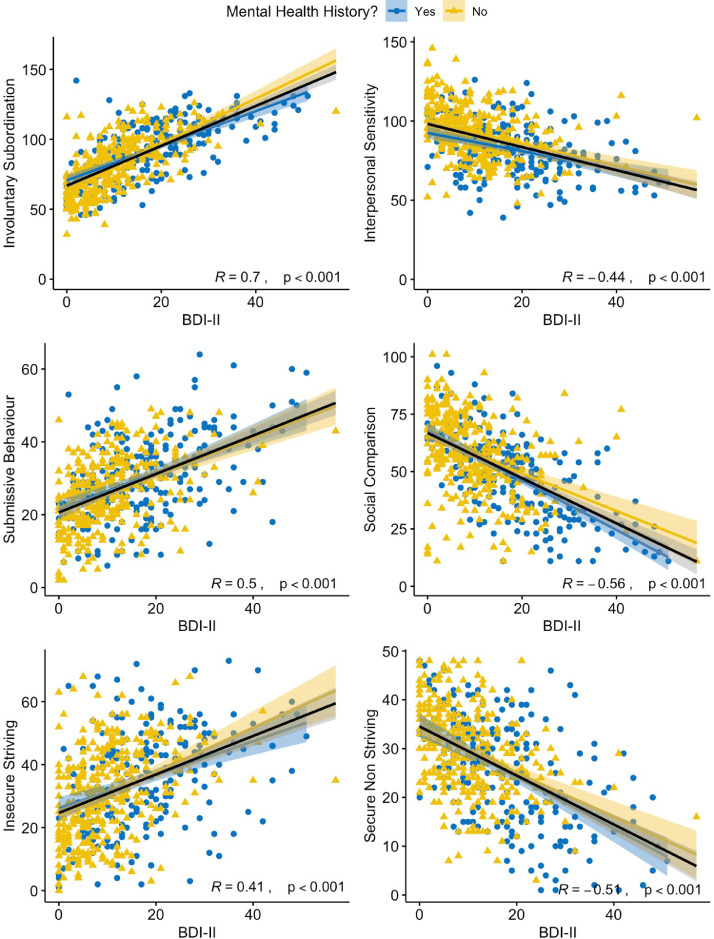
Correlations and effect sizes between depressive symptom severity, as measured by the BDI-II, and social measures, indicate significant relationships between low mood and social processes. Individuals with a history of mental health difficulties are indicated in blue (dots), those without in yellow (triangles). Overall regression fit and statistics are illustrated in black. Shaded error bands denote 95% confidence intervals.

Supplementary Table 2 (see Supplementary Materials) presents the descriptive statistics for each measure across the entire sample. Pearson correlations (controlling for gender) indicated that, as expected, there were significant positive associations between higher scores on the BDI-II and on all social measures (Fig. 1). In line with our hypothesis and both the Social Rank theory (Allan and Gilbert, 1997; Gilbert et al., 2007) and the Social Risk Hypothesis (Allen and Badcock, 2003), higher scores on the BDI-II were associated with feelings of lower social status and greater levels of submissive behaviour, involuntary subordination and inter-personal rejection sensitivity. See Supplementary Table 3 for a full correlation matrix of all measures.

### 3.2 Effects of self-reported history of mental health difficulties

Table 1 presents the scores on the affective and social measures for those with (“Yes”) and without mental health difficulties (“No”) and the ANCOVA results. Results revealed a significant main effect of group between those with and without a history of mental health difficulties across all affective and social measures. We conducted a final multivariate ANOVA to account for the variability across the social measures when determining between-group differences. Again, there was a significant main effect for group (F [6, 580] = 12.75, *p* < 0.001, Wilk's Λ = 0.88, η ^p2^ = 0.18).

**Table 1:** Univariate results for affective and social measures as a function of mental health difficulties.

Measure	n	Mental Health Difficulties	Mean	Std. Dev.	Range (Min-Max)	df	F	η^2^_p_
BDI-II	224	Yes	17.78	11	0–51	[1, 568]	84.96	.13
	348	No	10.1	8.15	0–57			
IPSM	230	Yes	82.3	16.72	39–126	[1, 590]	54.24	.084
	364	No	93.03	15.89	48–146			
SCS	230	Yes	48.64	17.66	11–96	[1, 589]	33.17	.053
	363	No	57.55	17	11–101			
SBS	230	Yes	30.41	11.78	6–64	[1, 590]	26.47	.043
	364	No	25.5	9.35	2–49			
ISQ	228	Yes	92.5	20.76	46–142	[1, 587]	48.5	.076
	363	No	80.61	18.94	32–126			
SAIS-I-IS	230	Yes	35.26	15.91	1–73	[1, 589]	12.1	.02
	363	No	30.82	14.25	0–68			
SAIS-I-SNS	230	Yes	25.49	11.01	1–48	[1, 590]	23.52	.038
	364	No	29.56	8.74	3–48			

*Note:*  BDI-II, Beck Depression Inventory; IPSM, Interpersonal Sensitivity Measure; ISQ, Involuntary Subordination Questionnaire; SAIS-I, Strive to Avoid Inferiority Scale Part I: IS – Insecure Striving, SNS – Secure Non-Striving; SBS, Submissive Behaviour Scale; SCS, Social Comparison Scale. All tests, variances of groups assumed equal and age and gender were included as covariates. All significant at *p* < 0.001.

## 4 Discussion

In a large community sample, we showed that self-reported current symptoms of depression positively correlated with subjective feelings of low status and submissive behaviour, in line with our hypothesis and with the previous literature (Allen and Badcock, 2003; Gilbert et al., 2009; O'Connor et al., 2002; Wood and Irons, 2015), though as the data is cross-sectional we were unable to ascertain any causal influence. Furthermore, participants with a self- reported history of mental health difficulties showed elevated depressive symptoms, increased levels of submissive behaviour and considered themselves as having low social status. The community sample reported a broad range of mental health problems, though depression was the problem most predominantly reported (~75%). However, we were unable to adjust for confirmed clinical diagnosis, whether participants were currently experiencing a depressive episode or not, and for medication effects. The nature of the data means that self-reported history of mental health problems and elevated current depression symptomatology are confounded. A case-control study, where current depressive symptom levels are matched across a sample with a history of depression that is currently in remission and a sample with no depression history (relative to a currently depressed group), would provide a more definitive evaluation of the relationships between depressive status and social processing that is unconfounded by residual symptom effects. That is the focus of Study 2.

## 5 Study 2: systemic biases in social, affective and cognitive processing in remitted depression

### 5.1 Aims

Using a remitted-participant adaptation of a case-control design, the aim of the second study was to investigate the systemic biases in social processing across three groups: individuals with a current diagnosis of clinical depression who are either in i) a current acute episode (‘Currently Depressed’ group) or ii) in clinical remission (‘Remitted Depressed’ group), versus iii) never-depressed controls. To address this question, we implemented the same battery of social measures outlined in the previous study, alongside well-established affective measures assessing for low mood. Critically, we sought to match current depressive symptoms across our remitted depressed and never-depressed groups, to disaggregate the effects of residual symptoms from depression history.

Our hypotheses were as follows;

• Depressed individuals currently in episode (‘Currently Depressed’ group) will show significantly higher affective symptoms and lower perceived social status, compared to Never-Depressed controls (Hypothesis 1).
• Remitted Depressed individuals will show significantly lower perceived social status, but will not show increased affective symptoms, relative to Never-Depressed individuals (Hypothesis 2).

We had no precise predictions regarding comparisons between the Currently Depressed and Remitted Depressed groups on social measures (although we expected the Currently Depressed group, by definition, to score higher on affective symptom measures).

### 5.2 Methods and materials

#### 5.2.1 Participants

Forty participants experiencing a current Major Depressive Episode (MDE) and meeting criteria for a diagnosis of Major Depressive Disorder (MDD) according to the DSM-IV (31:9 female:male; mean age: 36.38 ± 13.06 years) comprised our Currently Depressed group. Eighteen depressed participants who did not meet criteria for a current MDE but had previously experienced at least one MDE and met diagnostic criteria for MDD (13:5 female:male; mean age: 38.72 ± 13.06 years) formed our Remitted Depressed group. Depressive status for the Currently Depressed and Remitted Depressed participants was determined using the Structured Clinical Interview for the DSM-IV (First et al., 1995) (See Supplementary Methods for further details), administered by trained research staff under the supervision of a clinical psychologist. Finally, 64 healthy control participants who reported never meeting criteria for MDD (38:26 female:male; mean age: 33.16 ± 15.37 years) comprised our Never-Depressed Group. The Remitted Depressed group was matched on current depressive symptoms (indexed via the BDI-II) with the Never-Depressed group using sensitivity sampling, with 88.65% of the overall sample retained. This meant that any participants endorsing ‘zero’ on all items of the BDI-II were removed, as well as any Never-Depressed and Remitted Depressed participants scoring above 20, the clinical cut-off for moderate depression (Wang and Gorenstein, 2013). This ensured that both the Remitted Depressed and Never-Depressed groups had comparable levels of depressive symptoms, relative to the Currently Depressed group. All participants were recruited from volunteer panels at the University of Cambridge.

Table 2 shows the sample characteristics for the Never-Depressed, Currently Depressed and Remitted Depressed groups. Chi-squared analyses revealed no significant associations between group and descriptive measures. In addition, groups were evenly matched in their estimated premorbid verbal IQ according to the National Adult Reading Test – a commonly used measure to estimate premorbid verbal IQ levels in clinical samples (see Supplementary Materials). Preliminary sensitivity analyses addressing potential effects of gender revealed no differences in the pattern of results when including gender as a co-variate. The use of medication and range of dosages within the Remitted Depressed and Currently Depressed groups are presented in the Supplementary Materials (Supplementary Tables 4–5).

**Table 2:** Demographic characteristics. Numbers are ns unless otherwise stated.

	Never-Depressed (*n* = 64)	Currently Depressed (*n* = 40)	Remitted Depressed (*n* = 18)	F/ χ^2^	p value
Gender				3.91	0.14
Female	38 (59.4%)	31 (77.5%)	13 (72.2%)		
Male	26 (40.6%)	9 (22.5%)	5 (27.8%)		
Age, yrs				1.24	0.29
Means (SD)	33.16 (15.37)	36.38 (13.06)	38.72 (16.30)		
National Adult Reading Test				0.52	0.60
Means (SD)	10.00 (5.98)	8.92 (5.26)	10.40 (6.29)		
Ethnicity				12.48+	0.52
Caucasian	58 (90.6%)	38 (95.0%)	16 (88.9%)		
Black	2 (3.1%)	0 (0.0%)	0 (0.0%)		
Asian-Indian	1 (1.6%)	0 (0.0%)	0 (0.0%)		
Asian-Chinese	1 (1.6%)	0 (0.0%)	1 (5.6%)		
Mixed Asian-Caucasian	1 (1.6%)	0 (0.0%)	1 (5.6%)		
Mixed Black-Caucasian	1 (1.6%)	0 (0.0%)	0 (0.0%)		
Asian-Other	0 (0.0%)	0 (0.0%)	1 (4.5%)		
Hispanic	0 (0.0%)	1 (2.5%)	0 (0.0%)		
Mixed Other	0 (0.0%)	1 (2.5%)	0 (0.0%)		
Marital Status				8.61+	0.17
Single/Unmarried	46 (71.9%)	19 (47.5%)	9 (50.0%)		
Married	8 (12.5%)	10 (25.0%)	5 (27.8%)		
Separated/Divorced	5 (7.8%)	5 (12.5%)	1 (5.6%)		
Other	5 (7.8%)	6 (15.0%)	3 (16.7%)		
Education				15.01+	0.07
Completed Bachelor degree	25 (39.1%)	7 (17.5%)	4 (22.2%)		
Completed HSC	24 (37.5%)	14 (35.0%)	5 (27.8%)		
Completed Masters degree	7 (10.9%)	4 (10.0%)	2 (11.1%)		
Completed PhD	0 (0.0%)	3 (7.5%)	1 (5.6%)		
Completed Year 10	2 (3.1%)	4 (10.0%)	1 (5.6%)		
Other	6 (9.4%)	8 (20.0%)	5 (27.8%)		
Employment Status				5.48 +	0.47
Employed	41 (64.1%)	24 (60.0%)	7 (38.9%)		
Other	2 (3.1%)	3 (7.5%)	2 (11.1%)		
Student	5 (7.8%)	3 (7.5%)	2 (11.1%)		
Unemployed	16 (25.0%)	10 (25.0%)	7 (38.9%)		
Employment Type				6.37+	0.17
Full Time	19 (29.7%)	20 (50.0%)	5 (27.8%)		
Other	26 (40.6%)	13 (32.5%)	10 (55.6%)		
Part Time	19 (29.7%)	7 (17.5%)	3 (16.7%)		

Note: + denotes Fisher's exact test; HSC=Higher School Certificate.

#### 5.2.2 Social and affective measures

Participants were administered the same battery of measures as described in Study 1. See Supplementary Information for more details.

#### 5.2.3 Procedure

Following the clinical interview and an experimental task described elsewhere (Gillard et al., 2018), participants were administered the battery of social and affective self-report measures. Only data from these administered measures will be presented here. Participants were reimbursed for their time and the study was carried out in accordance with the Declaration of Helsinki and Good Clinical Practice and approved by the Cambridge Psychology Research Ethics Committee (PRE:2014.43).

#### 5.2.4 Data analysis

As in Study 1, missing data within and across different measures constituted less than 5% of the overall data, thus considered to be missing at random and the univariate ANOVAs therefore implemented listwise deletion, followed by a missing value analysis using EM (expectation-maximisation) (Dong and Peng, 2013). This method assumes a distribution for the partially missing data and bases inferences on the likelihood under that distribution, considering the conditions under which missing data occurred.

In order to examine the relationship between depressive status and social processing, we carried out a series of univariate ANCOVAs for the battery of social and affective measures with group as the between-group factor across all analyses. Planned contrasts were run to further examine differences between the Currently Depressed and Never-Depressed groups (Hypothesis 1) and between Remitted Depressed, relative to Never-Depressed groups (Hypothesis 2). To examine potential differences between Remitted Depressed and Currently Depressed groups, post-hoc comparisons were run between groups using a Bonferroni correction. Finally, a multivariate analysis was run, as in Study 1, to account for the variability across the social measures when determining between-group differences.

As in Study 1, we chose not to employ any data reduction techniques on the social measures.

## 6 Results

### 6.1 Social and affective measures

Table 3 presents the scores on the affective and social measures for our three groups. With the exception of the Secure Non-Striving Subscale (SNS) of the SAIS-I, results revealed a significant main effect of group between Remitted Depressed, Currently Depressed and Never-Depressed across all affective and social measures (see Table 3 for inferential statistics). There was no main effect of gender across all analyses.

**Table 3:** Descriptive and inferential statistics for affective and social measures across the three groups.

Measure	n	Group	Mean	Std Dev	df	F	Sig.	η^2^_p_
BDI-II	64	Never-Depressed*	7.41	4.31	[2118]	79.09	<0.001	0.57
	40	Currently Depressed*°	23.98	9.57				
	18	Remitted Depressed°	8.09	5.63				
IPSM	64	Never-Depressed*^+^	112.27	22.22	[2118]	27.73	<0.001	0.32
	40	Currently Depressed*	99.78	25.16				
	18	Remitted Depressed^+^	90.59	27.70				
ISQ	64	Never-Depressed*^+^	99.94	14.65	[2118]	44.72	<0.001	0.43
	40	Currently Depressed*°	120.65	13.07				
	18	Remitted Depressed^+^°	111.56	10.13				
SAIS-I-IS	64	Never-Depressed*^+^	108.44	16.44	[2118]	9.21	<0.001	0.14
	40	Currently Depressed*	71.86	17.01				
	18	Remitted Depressed^+^	103.14	15.91				
SAIS-I-SNS	64	Never-Depressed	91.51	14.96	[2118]	1.55	0.22	0.03
	40	Currently Depressed	85.01	21.68				
	18	Remitted Depressed	29.63	14.52				
SBS	64	Never-Depressed*^+^	41.83	17.19	[2118]	19.57	<0.001	0.25
	40	Currently Depressed*	39.31	9.54				
	18	Remitted Depressed^+^	35.06	15.85				
SCS	64	Never-Depressed*	24.78	10.64	[2118]	13.13	<0.001	0.18
	40	Currently Depressed*	21.78	8.62				
	18	Remitted Depressed	21.54	5.61				

*Note:* BDI-II, Beck Depression Inventory; IPSM, Interpersonal Sensitivity Measure; ISQ, Involuntary Subordination Questionnaire; SAIS-I Strive to Avoid Inferiority Scale Part I: IS – Insecure Striving, SNS – Secure Non-Striving; SBS, Submissive Behaviour Scale; SCS, Social Comparison Scale. All tests, variances of groups assumed equal. Analyses with gender and education included as a co-variate did not change the pattern above. ^+^Significant paired difference between Never-Depressed and Remitted Depressed.

⁎ Significant paired difference between Never-Depressed and Currently Depressed.°Significant paired difference between Remitted Depressed and Currently Depressed.

Planned contrast analyses broadly supported our first hypothesis that Currently Depressed participants would show significant differences on all social measures relative to Never-Depressed participants (all *p* < 0.001), apart from the SAIS-I Secure Non-Striving Subscale (*p* = 0.13). As also expected, the Currently Depressed group scored higher than the Never-Depressed participants on all the affective and symptom measures (See Table 3).

In line with Hypothesis 2, Remitted Depressed participants were *not* significantly different from Never-Depressed participants on the BDI-II (*p* = 0.70). Also in line with our second hypothesis, the Remitted Depressed participants were significantly different to Never-Depressed participants on the IPSM (*p* = 0.002), ISQ (*p* < 0.001), SAIS-I Insecure Striving Subscale (*p* = 0.02), and SBS (*p* = 0.03). The two groups were not significantly different, however, on the SCS (*p* = 0.19) or the SAIS-I Secure Non-Striving Subscale (*p* = 0.21).

Although we had no specific predictions, we also compared the Remitted Depressed and Currently Depressed samples. Unsurprisingly, the groups were significantly different on the affective measure, with the Currently Depressed group scoring higher on the BDI-II (p_bonf_ <0.001). Interestingly, however, the Currently Depressed participants were *not* significantly different from the Remitted Depressed group on the IPSM (p_bonf_ = 0.06), SBS (p_bonf_ = 0.06), SCS (p_bonf_ = 0.05) and both SAIS Insecure Striving (p_bonf_ = 1.00) and SAIS Secure Non-Striving Subscale (p_bonf_ = 1.00) subscales. The groups were however different on the ISQ (p_bonf_ = 0.04).

Replicating findings from Study 1, a multivariate analysis to account for the variability across the social measures when determining between-group differences revealed a significant main effect for group (F [2, 118] = 8.33, *p* < 0.001, Wilk's Λ = 0.54, η^2^_p_ = 0.27).

## 7 Discussion

We predicted that compared to Never-Depressed Controls, Currently depressed individuals would show evidence of maladaptive subordinate social processing strategies associated with a particular cognitive orientation to self and others. We further predicted that compared to the Never-Depressed Controls, Remitted Depressed individuals would continue to show evidence of being continually influenced by this strategy in the absence of current symptomatology.

In line with these predictions, results highlight specific orientations to rank-related submissive social processing strategies linked to mental health status. Currently Depressed participants reported enduring maladaptive submissive social strategies relative to Never Depressed controls and this defensive social strategy persisted in Remitted Depressed participants relative to Never-Depressed controls. Exploratory analyses revealed that the extent of these persistent maladaptive states in those in remission from depression was not significantly different to the currently depressed group across most measures.

Importantly, our findings extend the relationship between well-established links of involuntary subordination and the depressed states they give rise to, and systemic biases in social processing on a range of social-cognitive measures. More critically, we provide evidence of residual perceptions of low social status and submissiveness in a sample with a diagnosis of depression but currently in clinical remission, with an absence of current depressive symptomatology. In line with the predictions of the Social Risk Hypothesis of depression (Allen and Badcock, 2003), such a socio-affective profile may be a significant predisposing factor to risk of relapse and maintenance of Major Depressive Disorder. This may warrant further research to establish the mechanism of relapse in Remitted Depressed samples, for example, by longitudinally exploring whether maladaptive social states predict future relapse.

## 8 General discussion

As described within Social Rank Theory (Allan and Gilbert, 1997; Gilbert, 2000; Gilbert et al., 2007) and in line with the Social Risk Hypothesis (SRH) of depression (Allen and Badcock, 2003), depressed individuals with low perceptions of social rank are argued to employ a range of defensive behavioural strategies in response to internalised perceptions of inferiority and feelings of low self-esteem (Gilbert, 2000), with the overarching goal of maintaining inclusiveness in the group. Findings from the two studies reported here provide support for these views. Individuals with elevated self-reported depressive symptoms scores within a community sample (Study 1) as well as currently depressed individuals (Study 2) report experiencing significantly more low-rank self-perceptions, feelings of inferiority, as well as higher levels of involuntary subordination and submissiveness. These findings align with the existing literature, which identifies an association between negative social comparisons and increased rumination and depressive symptoms (Feinstein and Hershenberg, 2013), eating disorders (Troop et al., 2014), chronic illness (Terol et al., 2015), increased stigma in intellectual disability (Paterson et al., 2012) and increases in paranoid thoughts (Freeman et al., 2014).

We show for the first time that individuals with a diagnosis of depression but currently in clinical remission, and with comparable levels of self-reported depressive symptoms to never-depressed controls (Study 2) exhibited greater levels of submissive behaviour, involuntary subordination, interpersonal rejection sensitivity, and insecure striving to avoid inferiority. Social comparison also revealed lower average scores, equivalent to the Currently Depressed group, however these were not significantly different from the Never-Depressed groups. This provides support for the notion of a learned “lower rank” mind-set; in which systemic biases in social processing aimed at reducing risk in the short-term may initiate maladaptive behaviour in the long-term, even in the absence of current depressed mood. Several lines of evidence point to the experience social defeat and loss of resources as predictors of depressed mood rather than socially subordinate behaviour in general (Gilbert et al., 2009; Taylor et al., 2011; Larrieu and Sandi, 2018). The persistence of submissive and subordinate behaviours may therefore be a maladaptive protective mechanism to inhibit further loss and defeat whilst at the same time conferring risk of depressive relapse in line with the predictions of the Social Risk Hypothesis (Allen and Badcock, 2003; 2006)

To the best of our knowledge, this study validates the sensitivity of the Involuntary Subordination Questionnaire (Sturman et al., 2011) in a clinically depressed sample for the first time, however, our study has several limitations. We were unable to discern historical or current clinical diagnosis in the community sample of Study 1, as we were reliant on self-report. Although evidence for the validity of the BDI-II and BAI as reliable indicators of symptom severity and as screening tools within the general population has been reported (Beck et al., 1997; Muntingh et al., 2011; Osman et al., 2002; Storch et al., 2004), this limits the interpretation of analyses to more precise clinical groups. Furthermore, we were unable to disentangle the effects of other symptoms of psychopathology, such as anxiety and paranoia and medication on perception of social status across both studies and this remains an important area for future research. Finally, longitudinal designs to explore the causal relationship between social defeat and the onset of depressive symptoms are desirable.

In sum, currently depressed individuals were found to report greater sensitivity to social rejection, and engagement in negative social comparisons, submissive behaviour and involuntary subordination, while striving to avoid inferiority. Importantly, we show for the first time that this profile is maintained in depressed individuals’ currently in clinical remission and in the absence of acute depressed mood (Study 2) as well as in individuals with self-identified mental health difficulties in a community sample (Study 1). These findings suggest that evolutionarily-rooted submissive strategies of social affiliation can become maladaptive and may confer increased vulnerability to future depressive episodes to those in remission. These findings contribute to a scientific rationale for incorporating functional treatment outcomes from within the social domains into existing prevention approaches in depression.

## Contributors

JG, TD and JS developed the study concept and design. Testing and data collection were performed by JG, SG, KG, CH, JS. JG and JS performed the data analysis. JG, TD and JS drafted the paper. All authors approved the final version of the paper for submission.

## Funding statement

This work was funded by the UK Medical Research Council (Grant Reference: SUAG/043 G101400) and supported by the National Institute for Health Research Cambridge Biomedical Research Centre.

## Data availability statement

Data is available upon request from the corresponding author.

Author Statement

## Declaration of Competing Interest

All authors declare no conflict of interest.

## Appendix Supplementary materials

Image, application 1
