# Supplementary material for: Converging evidence for enduring perceptions of low social status in individuals in remission from depression
Source: J Affect Disord. 2021 Nov 1;294:661–70. doi: 10.1016/j.jad.2021.07.083 (PMC8411663; doi:10.1016/j.jad.2021.07.083)
Supplement: Supplementary file 1 [file mmc1.docx]

# Supplementary Material

**Title**: Converging evidence for enduring perceptions of low social status in individuals in remission from depression.

**Running Title**: Social difficulties in remitted depression

**Authors:** Julia A. Gillard^a^, Siobhan Gormley^a^, Kirsty Griffiths^a^, Caitlin Hitchcock^a^, Tim Dalgleish*^a, b^, Jason Stretton*^a^.

**Institution**:

^1^Medical Research Council Cognition and Brain Sciences Unit, University of Cambridge, Cambridge, CB2 7EF, United Kingdom

^2^Cambridgeshire and Peterborough NHS Foundation Trust

^*^Should be considered as joint senior authors

**Corresponding Author:** *Jason Stretton,* MRC Cognition and Brain Sciences Unit, 15 Chaucer Road, CB2 7EF, Cambridge, United Kingdom. [jason.stretton@mrc-cbu.cam.ac.uk](mailto:jason.stretton@mrc-cbu.cam.ac.uk)

# Study 1: Supplementary Methods

**Supplementary Table 1.** Demographic characteristics for the sample in Study 1. All numbers are n unless stated otherwise.

|  | **Mental Health Difficulties?** | |  |  | |  |
| --- | --- | --- | --- | --- | --- | --- |
|  | **Yes (n=232)** | **No (n=368)** | **Total (N=600)** | **F/ χ2** | **p** |  |
| Gender |  |  |  | 6.24+ | 0.04 |  |
| Male | 65 (28.0%) | 133 (36.2%) | 198 (33.1%) |  |  |  |
| Female | 164 (70.7%) | 233 (63.5%) | 397 (66.3%) |  |  |  |
| Other | 3 (1.3%) | 1 (0.3%) | 4 (0.7%) |  |  |  |
| Age, yrs |  |  |  | 3.74 | 0.05 |  |
| Mean (SD) | 39.92 (13.57) | 42.08 (13.10) | 41.24 (13.32) |  |  |  |
| Ethnicity |  |  |  | 11.53+ | 0.64 |  |
| White-British | 166 (92.2%) | 273 (91.9%) | 439 (92.0%) |  |  |  |
| White-Irish | 0 (0.0%) | 2 (0.7%) | 2 (0.4%) |  |  |  |
| White-Gypsy or Irish Traveller | 0 (0.0%) | 0 (0.0%) | 0 (0.0%) |  |  |  |
| White-Other | 4 (2.2%) | 5 (1.7%) | 9 (1.9%) |  |  |  |
| Mixed-White & Black Caribbean | 1 (0.6%) | 2 (0.7%) | 3 (0.6%) |  |  |  |
| Mixed-White & Black African | 1 (0.6%) | 3 (1.0%) | 4 (0.8%) |  |  |  |
| Mixed-White & Asian | 1 (0.6%) | 0 (0.0%) | 1 (0.2%) |  |  |  |
| Mixed -Other | 0 (0.0%) | 0 (0.0%) | 0 (0.0%) |  |  |  |
| Asian-Indian | 2 (1.1%) | 1 (0.3%) | 3 (0.6%) |  |  |  |
| Asian-Pakistani | 0 (0.0%) | 2 (0.7%) | 2 (0.4%) |  |  |  |
| Asian-Bangladeshi | 1 (0.6%) | 1 (0.3%) | 2 (0.4%) |  |  |  |
| Asian-Chinese | 0 (0.0%) | 3 (1.0%) | 3 (0.6%) |  |  |  |
| Asian-Other | 0 (0.0%) | 0 (0.0%) | 0 (0.0%) |  |  |  |
| African | 2 (1.1%) | 2 (0.7%) | 4 (0.8%) |  |  |  |
| Caribbean | 0 (0.0%) | 2 (0.7%) | 2 (0.4%) |  |  |  |
| Other Black/African/Caribbean | 1 (0.6%) | 0 (0.0%) | 1 (0.2%) |  |  |  |
| Arab | 0 (0.0%) | 1 (0.3%) | 1 (0.2%) |  |  |  |
| Other ethnic group | 1 (0.6%) | 0 (0.0%) | 1 (0.2%) |  |  |  |
| Nationality |  |  |  | 5.03+ | 0.29 |  |
| British | 180 (77.6%) | 298 (81.0%) | 478 (79.7%) |  |  |  |
| American | 27 (11.6%) | 31 (8.4%) | 58 (9.7%) |  |  |  |
| Irish | 5 (2.2%) | 16 (4.3%) | 21 (3.5%) |  |  |  |
| Canadian | 16 (6.9%) | 17 (4.6%) | 33 (5.5%) |  |  |  |
| Australian | 4 (1.7%) | 6 (1.6%) | 10 (1.7%) |  |  |  |
| Education |  |  |  | 3.53+ | 0.61 |  |
| UK GCSE's or equivalent | 35 (15.3%) | 58 (15.9%) | 93 (15.7%) |  |  |  |
| UK A level's or equivalent | 57 (24.9%) | 98 (26.8%) | 155 (26.1%) |  |  |  |
| Bachelors degree | 85 (37.1%) | 147 (40.3%) | 232 (39.1%) |  |  |  |
| Master's degree | 34 (14.8%) | 38 (10.4%) | 72 (12.1%) |  |  |  |
| PhD | 6 (2.6%) | 10 (2.7%) | 16 (2.7%) |  |  |  |
| Other | 12 (5.2%) | 14 (3.8%) | 26 (4.4%) |  |  |  |
| Employment Status |  |  |  | 6.25+ | 0.18 |  |
| Unemployed | 31 (13.5%) | 40 (11.0%) | 71 (12.0%) |  |  |  |
| Student | 21 (9.2%) | 27 (7.4%) | 48 (8.1%) |  |  |  |
| Employed Part time | 43 (18.8%) | 72 (19.8%) | 115 (19.4%) |  |  |  |
| Employed Full time | 91 (39.7%) | 176 (48.4%) | 267 (45.0%) |  |  |  |
| Other | 43 (18.8%) | 49 (13.5%) | 92 (15.5%) |  |  |  |
| *Note*: + denotes Fisher’s exact test. GCSE= General Certificate of Secondary Education. A Level=Advanced Level | | | | | | |

# Study 1: Supplementary Results

Supplementary Table 2. Mean scores for all affective and social measures across the whole sample in Study 1.

|  | **n** | | **Range** | **Min.** | **Max.** | **Mean** | **Std. Deviation** |
| --- | --- | --- | --- | --- | --- | --- | --- |
| BDI-II | | 578 | 57 | 0 | 57 | 13.05 | 10.05 |
| IPSM | | 600 | 107 | 39 | 146 | 88.88 | 16.97 |
| SCS | | 599 | 90 | 11 | 101 | 54.14 | 17.75 |
| SBS | | 600 | 62 | 2 | 64 | 27.39 | 10.59 |
| ISQ | | 597 | 110 | 32 | 142 | 85.14 | 20.4 |
| SAIS-I-IS | | 599 | 73 | 0 | 73 | 32.58 | 15.02 |
| SAIS-I-SNS | | 600 | 47.00 | 1.00 | 48 | 28 | 9.85 |

Note; BDI-II, Beck Depression Inventory; IPSM, Interpersonal Sensitivity Measure; ISQ, Involuntary Subordination Questionnaire; SAIS-I, Striving to Avoid Inferiority Scale Part I: IS – Insecure Striving, SNS – Secure Non-Striving; SBS, Submissive Behaviour Scale; SCS, Social Comparison Scale.

**Supplementary Table 3.** Partial correlation matrix (1-tailed) of all social and affective measures, adjusting for gender in Study 1.

|  | | | BDI-II | SCS | SBS | ISQ | IPSM | SAIS-IS |
| --- | --- | --- | --- | --- | --- | --- | --- | --- |
|  | BDI-II | Correlation | 1.00 |  |  |  |  |  |
|  |  | Significance | . |  |  |  |  |  |
|  |  | df | 0 |  |  |  |  |  |
|  | SCS | Correlation | -.56 | 1.00 |  |  |  |  |
|  |  | Significance | .00 | . |  |  |  |  |
|  |  | df | 569 | 0 |  |  |  |  |
|  | SBS | Correlation | .50 | -.47 | 1.00 |  |  |  |
|  |  | Significance | .00 | .00 | . |  |  |  |
|  |  | df | 569 | 569 | 0 |  |  |  |
|  | ISQ | Correlation | .70 | -.50 | .71 | 1.000 |  |  |
|  |  | Significance | .00 | .00 | .00 | . |  |  |
|  |  | df | 569 | 569 | 569 | 0 |  |  |
|  | IPSM | Correlation | -.44 | .41 | -.62 | -.57 | 1.00 |  |
|  |  | Significance | .00 | .00 | .00 | .00 | . |  |
|  |  | df | 569 | 569 | 569 | 569 | 0 |  |
|  | SAIS-IS | Correlation | .42 | -.34 | .50 | .60 | -.49 | 1.00 |
|  |  | Significance | .00 | .00 | .00 | .00 | .00 | . |
|  |  | df | 569 | 569 | 569 | 569 | 569 | 0 |
|  | SAIS- SNS | Correlation | -.52 | .52 | -.44 | -.57 | .46 | -.66 |
|  |  | Significance | .00 | .00 | .00 | .00 | .00 | .00 |
|  |  | df | 569 | 569 | 569 | 569 | 569 | 569 |

Note; BDI-II, Beck Depression Inventory; IPSM, Interpersonal Sensitivity Measure; ISQ, Involuntary Subordination Questionnaire; SAIS-, Striving to Avoid Inferiority Scale Part I: IS – Insecure Striving, SNS – Secure Non-Striving; SBS, Submissive Behaviour Scale; SCS, Social Comparison Scale.

# Study 2: Supplementary Methods

*Participant Co-morbidity and Medication Use*

Participants were excluded if they had a diagnosis of psychosis, bipolar disorder, current or past drug or alcohol problems, or a personality disorder. Moreover, the co-occurrence of multiple psychological disorders (comorbidity) is a common problem when recruiting clinical participants, with 7.7% and 17.3% lifetime prevalence for 2 or more and 3 or more disorders, respectively (Kessler et al., 2005, 2012). However, in line with the existing literature, it would be unfeasible to exclude all participants with additional diagnoses if these are not thought to impact largely on the phenomenon of interest. See Supplementary Table 4 for information regarding comorbidities. In addition, in our sample, 44% of the Currently Depressed group reported currently using medication, compared to 26% of the Remitted Depressed sample. The use of medication and range of dosages within the Remitted Depressed and Currently Depressed sample are presented below. Never-Depressed participants indicated no current or past use of antidepressant medication. See Supplementary Table 5.

Supplementary Table 4. Co-morbidities (*n*s) in the Currently Depressed group (n=40) in Study 2

| **Co-morbidity** | **Current** | **Past** |
| --- | --- | --- |
| Generalized Anxiety Disorder | 7 | 1 |
| Post-traumatic Stress Disorder | 2 | 2 |
| Obsessive-Compulsive Disorder | 2 | 1 |
| Social Anxiety Disorder | 2 | 1 |
| Panic Disorder | 2 | 2 |
| Eating Disorder | 0 | 2 |
| Total | 15 | 9 |

Supplementary Table 5. Medication use in the Currently Depressed and Remitted Depressed groups in Study 2

|  | Remitted Depressed  (n=18) | Currently Depressed (n = 40) | Total | Min Dosage (mg) | Max Dosage (mg) |
| --- | --- | --- | --- | --- | --- |
| Citalopram | 2 | 5 | 7 | 10 | 40 |
| Venlafaxine | 2 | 2 | 4 | 150 | 375 |
| Fluoxetine | 0 | 5 | 5 | 20 | 60 |
| Prozac | 1 | 1 | 2 | 40 | 40 |
| Mirtazapine | 0 | 2 | 2 | 15 | 30 |
| Sertraline | 0 | 3 | 3 | 100 | 100 |
| Other | 0 | 2 | 2 | - | - |
| Total | 5 | 20 | 25 |  |  |

*Affective and Diagnostic Measures*

### Structured Clinical Interview for DSM-IV Axis- I Disorders (SCID-I; First et al., 1995).

The SCID-I is a standardized diagnostic interview schedule designed to assist clinicians and researchers in making reliable DSM-IV Axis I psychiatric diagnoses. The SCID-I involves a series of questions concerning current and past symptoms of a range of psychological disorders and usually takes between ½ and 1 hour. The SCID is only administered by experienced research staff that has undergone comprehensive SCID training. The mood module is used to verify whether participants are currently experiencing low mood of clinical severity or not. In this study, Currently Depressed participants previously underwent a structural clinical interview (SCID-I) to confirm their diagnosis and current episode.

### National Adult Reading Test (NART; Nelson, 1982).

The NART consists of participants reading aloud 50 difficult-to-pronounce words and is scored based on pronunciation errors made. The NART is widely used in clinical and research settings to estimate a person’s premorbid level of intellectual ability as a function of verbal intelligence, in neuropsychological research (Bright, Jaldow, & Kopelman, 2002). Moreover, Nelson & O’Connell (1978) showed the NART to be a robust predictor of premorbid levels on the Wechsler Adult Intelligence Scale (WAIS; Webster & Wechsler, 1958), suggesting that it has high construct validity as a measure of general intelligence, even when used in populations with neurological or psychiatric disorders (Crawford, Stewart, Cochrane, Parker, & Besson, 1989; Starr et al., 2004). A study comparing participants’NART scores at age 80 with intelligence scores collected at age 11 demonstrated the robustness of the NART at estimating verbal IQ across the lifespan (Starr et al., 2004). In the present study, the NART was used to measure verbal IQ and to enable matching across groups.

**Supplementary References**

Bright, P., Jaldow, E., & Kopelman, M. D. (2002). The National Adult Reading Test as a measure of premorbid intelligence: A comparison with estimates derived from demographic variables. *Journal of the International Neuropsychological Society*, *8*(6), 847–854. https://doi.org/10.1017/S1355617702860131

Crawford, J. R., Stewart, L. E., Cochrane, R. H. B., Parker, D. M., & Besson, J. A. O. (1989). Construct validity of the National Adult Reading Test: a factor analytic study. *Personality and Individual Differences*, *10*(5), 585–587. https://doi.org/10.1016/0191-8869(89)90043-3

First, M. B., Spitzer, R. L., Gibbon, M., Williams, J. B. W., Davies, M., Borus, J., … Rounsaville, B. (1995). *The Structured Clinical Interview for DSM-IV Axis I Disorders-Patient Edition*. *Biometrics Research Department*. https://doi.org/10.1521/pedi.1995.9.2.92

Kessler, R. C., Berglund, P., Demler, O., Jin, R., Merikangas, K. R., & Walters, E. E. (2005). Lifetime Prevalence and Age-of-Onset Distributions of. *Arch Gen Psychiatry*, *62*(June), 593–602. https://doi.org/10.1001/archpsyc.62.6.593

Kessler, R. C., Berglund, P., Demler, O., Jin, R., Merikangas, K. R., & Walters, E. E. (2012). Lifetime prevalence and age-of-onset distributions of DSM-IV disorders in the National Comorbidity Survey Replication. *Arch Gen Psychiatry*, *62*(June 2005), 593–602.

Nelson, H E. (1982). *National Adult Reading Test (NART): For the Assessment of Premorbid Intelligence in Patients with Dementia: Test Manual*. *1982*.

Nelson, Hazel E, & O’Connell, A. (1978). Dementia: The Estimation of Premorbid Intelligence Levels Using the New Adult Reading Test. *Cortex*, *14*(2), 234–244. https://doi.org/10.1016/S0010-9452(78)80049-5

Starr, J. M., McGurn, B., Whiteman, M., Pattie, A., Whalley, L. J., & Deary, I. J. (2004). Life long changes in cognitive ability are associated with prescribed medications in old age. *International Journal of Geriatric Psychiatry*, *19*(4), 327–332. https://doi.org/10.1002/gps.1093

Webster, A. S., & Wechsler, D. (1958). The Measurement and Appraisal of Adult Intelligence. *The Journal of Criminal Law, Criminology, and Police Science*, *49*(4), 362. https://doi.org/10.2307/1141601
